# Supplementary material for: Hydroxyurea maintains working memory function in pediatric sickle cell disease
Source: PLoS One. 2024 Jun 27;19(6):e0296196. doi: 10.1371/journal.pone.0296196 (PMC11210848; doi:10.1371/journal.pone.0296196)
Supplement: S1 Table — One-sample t-test or Wilcoxon signed rank test was used to compare 1-year follow-up to baseline; p indicates significance (two-sided). Table was regenerated with permission from Wang et al. (2021). (DOCX) [file pone.0296196.s001.docx]

**Supporting Information**

**S1 Table. Clinical hematologic values of the HU group at baseline and 1-year follow-up.** One-sample *t-*test or Wilcoxon signed rank test was used to compare 1-year follow-up to baseline; *p* indicates significance (two-sided). Table was regenerated with permission from Wang et al. (2021).

| **Variable (mean [SD])** | **Baseline** | **1-year follow-up** | **p-value** |
| --- | --- | --- | --- |
| **Hemoglobin (g/dl)** | 8.7 (1.2) | 10.1 (1.5) | .002 |
| **HbF (%)** | 8.5(5.2) | 23.1 (5.7) | <.001 |
| **Absolute reticulocyte count (×10^3^/μl)** | 260,000 (60,000) | 140,000 (70,000) | <.001 |

Reference:

Wang WC, Zou P, Hwang SN, Kang G, Ding J, Heitzer AM, et al. Effects of hydroxyurea on brain function in children with sickle cell anemia. Pediatr Blood Cancer. 2021;68. doi:10.1002/PBC.29254
