# Supplementary material for: Hydroxyurea maintains working memory function in pediatric sickle cell disease
Source: PLoS One. 2024 Jun 27;19(6):e0296196. doi: 10.1371/journal.pone.0296196 (PMC11210848; doi:10.1371/journal.pone.0296196)
Supplement: S2 Table — (DOCX) [file pone.0296196.s002.docx]

**Supporting Information**

**S2 Table. Seven clusters (peak accuracy ≥.7 & volume ≥520 mm^3^) were selected from the results of searchlight analysis of the non-HU control group for downstream analysis.**

| **Cluster ID** | **Peak (x,y,z)** | **Peak accuracy** | **Cluster mean** | **Volume (mm^3^)** | **Brain regions with  % coverage in AAL** |
| --- | --- | --- | --- | --- | --- |
| 1 | 38, -82, -18 | 0.72 | 0.67 | 2496 | 44.87% Cerebelum_Crus1_R  23.08% Occipital_Inf_R  12.50% Temporal_Inf_R  8.01% Fusiform_R  6.41% Cerebelum_6_R |
| 2 | 16, -68, 64 | 0.72 | 0.67 | 1400 | 96.00% Parietal_Sup_R |
| 3 | 58, -50, 46 | 0.73 | 0.67 | 1072 | 82.84% Parietal_Inf_R  14.93% Angular_R |
| 4 | 64, -26, -28 | 0.70 | 0.66 | 704 | 52.27% Temporal_Inf_R  47.73% no_label |
| 5 | -12, -88, -24 | 0.74 | 0.68 | 648 | 83.95% Cerebelum_Crus1_L 11.11% Cerebelum_Crus2_L |
| 6 | 44, -74, 36 | 0.71 | 0.67 | 584 | 79.45% Angular_R 20.55% Occipital_Mid_R |
| 7 | -2, -94, 20 | 0.71 | 0.67 | 520 | 78.46% Cuneus_L 21.54% Calcarine_L |
