# Supplementary material for: Hydroxyurea maintains working memory function in pediatric sickle cell disease
Source: PLoS One. 2024 Jun 27;19(6):e0296196. doi: 10.1371/journal.pone.0296196 (PMC11210848; doi:10.1371/journal.pone.0296196)
Supplement: S3 Table — (DOCX) [file pone.0296196.s003.docx]

**Supporting Information**

**S3 Table. Two clusters (peak accuracy ≥.7 & volume ≥520 mm^3^) were selected from the results of searchlight analysis of the HU group for downstream analysis.**

| **Cluster ID** | **Peak (x, y, z)** | **Peak accuracy** | **Cluster mean** | **Volume (mm^3^)** | **Brain regions with % of coverage in AAL** |
| --- | --- | --- | --- | --- | --- |
| 1T | 24, 56, 4 | 0.72 | 0.67 | 1744 | 75.69% Frontal_Sup_2_R  24.31% Frontal_Mid_2_R |
| 2T | -32, 58, 2 | 0.71 | 0.66 | 1368 | 53.80% Frontal_Mid_2_L  44.44% Frontal_Sup_2_L |
